# DIAGNOSIS OF ACUTE APPENDICITIS AND APPENDICULAR PERFORATION: EVALUATION OF PLATELET INDICES AND RED CELL DISTRIBUTION WIDTH AS EMERGING BIOMARKERS

**DOI:** 10.1590/0102-672020230039e1757

**Published:** 2023-09-15

**Authors:** Chetan AG, Vijaya PATIL

**Affiliations:** 1 Shri B M Pati Medical College Hospital and Research Center, General Surgery – Vijayapura, Karnataka, Índia.

**Keywords:** Appendicitis, Erythrocyte Indices, Mean Platelet Volume, Apendicite, Índices de Eritrócitos, Volume Plaquetário Médio

## Abstract

**BACKGROUND:**

Acute appendicitis is a common surgical emergency worldwide. Recent studies on hematological inflammatory markers concerning acute appendicitis have shown variable results.

**AIMS:**

The aim of this study was to evaluate pre-operative values of platelet indices such as mean platelet volume (MPV) and platelet distribution width (PDW), and red cell distribution width (RDW) in relation to the diagnosis of acute appendicitis and their efficacy as predictors of appendicular perforation.

**METHODS:**

A prospective observational study of 190 patients diagnosed with appendicitis and who underwent an appendectomy was undertaken and confirmed histopathologically. Preoperatively, blood samples of white blood cells (WBCs), platelet count, MPV, PDW, and RDW were analyzed using a Sysmex XN1000 analyzer machine.

**RESULTS:**

Of 190 patients, 169 had acute appendicitis, and 21 had perforated appendicitis. The mean age of patients was 28.04 ± 14.2 years. The male-to-female ratio was 1.5:1. The WBC (p<0.05), MPV (p<0.05), and PDW (p<0.05) were found to have higher statistically significant values in acute appendicitis and perforated appendicitis compared to the RDW (p>0.05). However, perforated appendicitis had a higher RDW value compared to acute appendicitis, which can be a predictive factor.

**CONCLUSIONS:**

The elevated value of MPV and PDW associated with leukocytosis can be used as supportive evidence for the clinical and radiological diagnosis of acute appendicitis and appendicular perforation. Thus, these values can be used as diagnostic cost-effective inflammatory biomarkers.

## RESUMO

**RACIONAL:**

A apendicite aguda é uma emergência cirúrgica comum em todo o mundo. Estudos recentes sobre marcadores inflamatórios hematológicos relacionados à apendicite aguda mostraram resultados variáveis.

**OBJETIVOS:**

Avaliar valores pré-operatórios de índices plaquetários como volume médio de plaquetas (VPM) e largura de distribuição de plaquetas (PDW), largura de distribuição de hemácias (RDW) em relação ao diagnóstico de apendicite aguda e sua eficácia como preditores de apendicite perfuração.

**MÉTODOS:**

Estudo observacional prospectivo de 190 pacientes diagnosticados com apendicite e submetidos a apendicectomia confirmados histopatologicamente. Amostras de sangue pré-operatórias de glóbulos brancos (WBC), contagem de plaquetas, volume plaquetário médio (MPV), distribuição das plaquetas (PDW) e distribuição dos glóbulos vermelhos (RDW) foram analisadas usando uma máquina analisadora Sysmex XN1000.

**RESULTADOS:**

Foram incluídos 190 pacientes, sendo que 169 tiveram apendicite aguda e 21 tiveram apendicite perfurada. A média de idade dos pacientes foi de 28,04 ± 14,2. A proporção homem-mulher foi de 1,5:1. Verificou-se que WBC (p<0,05), MPV (p<0,05) e PDW (p<;0,05) têm valores estatisticamente significativos mais altos na apendicite aguda e na apendicite perfurada em comparação com o RDW (p> 0,05). No entanto, a apendicite perfurada apresentou um valor de RDW maior em comparação com a apendicite aguda, o que pode ser um fator preditivo.

**CONCLUSÕES:**

O valor elevado de MPV e PDW associado à leucocitose pode ser usado como evidência de suporte para o diagnóstico clínico e radiológico de apendicite aguda e perfuração apendicular. Assim, esses valors podem ser usado como biomarcadores inflamatórios diagnósticos de baixo custo.

## INTRODUCTION

Appendicectomy is the most common procedure done in a surgical emergency. Differential diagnosis of acute appendicitis is still a matter of concern in some atypical cases where delayed or inaccurate diagnosis may lead to several complications. Radiological imaging that helps in the surgical decision may not confirm the diagnosis or be unavailable in some hospitals. To prevent complications such as perforation and subsequent peritonitis, whether to operate or wait it out in patients with appendicitis remains a conundrum until the diagnosis is certain. Negative appendicectomy may lead to unnecessary morbidity and social burden risk due to improper symptom interpretation and early surgery^13^. Consequently, a prompt and precise diagnosis is essential. Surgeons regularly use the complete blood count (CBC) test, which is one of the most commonly used tests in clinical laboratories, and in the emergency room as part of the routine pre-operative evaluation and to identify inflammatory diseases. The white blood cell (WBC) count and neutrophil count are the early markers of inflammation in acute appendicitis^20^. However, depending on the population being investigated, the severity of the symptoms, and the cutoff values being employed, their sensitivity and specificity can vary greatly^3,22^.

The red cell distribution width (RDW) quantifies red blood cell size variability. RDW has been linked to the identification of a variety of inflammatory diseases, including acute appendicitis, according to studies. The erythrocyte index, a biological marker of inflammation that has been utilized in hematological practice, is currently accepted^18^. The elevated erythrocyte sedimentation rate and interleukin-6 levels are related to high RDW^16,18^.

The involvement of platelets in inflammation is critical. The function of platelets in the etiology of numerous disorders when inflammation occurs has been demonstrated in numerous studies. The coagulation pathway, severe infection, trauma, systemic inflammatory syndrome, and platelet disorders have all been linked to variations in platelet indices in these investigations^18^. Platelet indices, including mean platelet volume (MPV) and platelet distribution width (PDW), are indicators of platelet activation and are connected to platelet morphology.The MPV is the average size of the platelet in peripheral blood^15^. The PDW represents the variability in platelet size (anisocytosis) and heterogeneity in platelet morphology. An increase in PDW is caused by a change in platelet shape from discoid to spherical with pseudopod formation, which occurs during platelet activation^15^.

Many recent studies on hematological inflammatory markers in establishing the diagnosis of acute appendicitis are variable and debatable. Hence, we intended to study using basic, inexpensive, readily available, and convenient inflammatory markers presented in CBC to evaluate their value in establishing the diagnosis of acute appendicitis and its role in predicting appendicular perforation.

## METHODS

This was a prospective study conducted from January 2021 to November 2022, in the Department of General Surgery, Shri B M Patil Medical College and Hospital, and was approved by the Shri B M Patil Medical College and Hospital Ethical Committee (IEC/NO-09/2021).

The diagnosis of acute appendicitis was made by thorough clinical examination, appropriate laboratory, and radiological investigations. Venous blood samples were collected in di-potassium ethylenediaminetetraacetic acid tubes. The samples were run within 2 h of venipuncture using the 6-part differentiated automated hematoanalyzer (Sysmex XN-1000), and a CBC analysis of the samples, including the platelet indices (i.e., MPV, PDW, and PCT), was performed. A pretested structural pro forma was used to collect relevant information for each individual patient.

All patients who underwent appendicectomy were included in the study. All patients who were diagnosed with hemolytic disease, bleeding, and platelet disorder and also who were treated with drugs that alter coagulation profile and platelet count were excluded from the study.

### Statistics

The Statistical Package for the Social Sciences 23.0 package program was used in the statistical analysis of data. Categorical measurements were summarized as numbers and percentages, and continuous measurements as mean and standard deviation (median and minimum-maximum where necessary). The Mann-Whitney U test was used for parameters without normal distribution. In the case of unequal data distribution, the Kruskal-Wallis test (nonparametric independent-group comparison) was used to compare groups. In the study, the cutoff value was determined by calculating the sensitivity and specificity values and examining the area under the receiver operating characteristic (ROC) curve. p-value was considered non-significant at a level of >0.05 and significant at a level of <0.05.

## RESULTS

A total of 190 patients were enrolled in this study with a pre-operative diagnosis of appendicitis. Out of 190 patients, 116 (61%) were males and 74 (39%) were females, with a male-to-female ratio of 1.5:1. The ages of patients are ranging from 6 to 72 years with a mean of 28.04 ± 14.2 years.

Based on the histopathological report, 169 patients had acute appendicitis and 21 patients had perforated appendicitis. The comparison of demographic and blood investigation values between the groups is shown in Table 1.

**Table 1:** Distribution of mean of various hematological parameters between the acute appendicitis and perforated appendicitis

Parameters	Acute (n=169)		Perforated (n=21)		p-value
	Mean	±SD	Mean	±SD	
Total count	10475.62	3846.59	13947.19	7376.32	0.026^*^
Platelet count	283.89	70.19	311.33	154.37	0.440
PDW	11.60	1.91	10.92	2.47	0.036^*^
MPV	9.27	1.06	8.47	1.26	0.002^*^
RDW	14.71	1.64	15.61	2.81	0.442

^*^Significant at 5% level of significance (p<0.05). PDW: platelet distribution width; MPV: mean platelet volume; RDW: red cell distribution width; SD: standard deviation.

The parameters such as leukocyte count, PDW, and MPV were statistically significant, but platelet count and RDW were insignificant when compared with the two groups. However, leukocyte count and RDW showed higher values in the perforated group than in the acute group. But PDW and MPV showed a significantly decreased value in the perforated group than the acute group, which signifies the chronicity of the disease.

**Figure 1.**
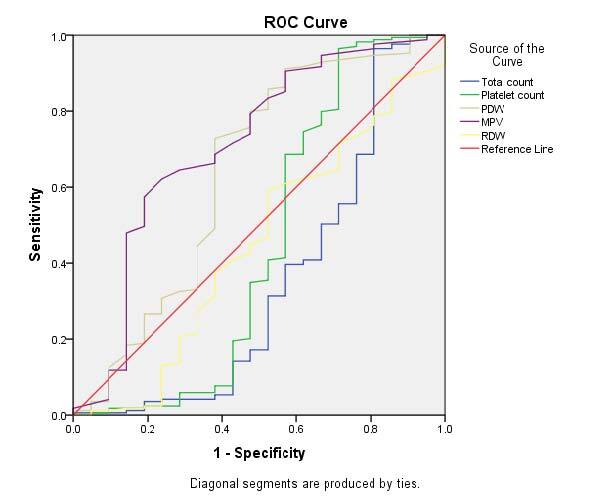
Receiver operating characteristic curve analysis showing all parameters in predicting acute appendicitis.

The diagnostic parameter value was analyzed with ROC analysis (Figure 1). Leukocyte count, PDW, and RDW were sensitive parameters, and MPV was the specific parameter for predicting acute and perforated appendicitis. The PDW and MPV level cutoff points for diagnosing and predicting perforation were <10.4 and <9.05 fl, respectively (Table 2).

**Table 2:** Receiver operating characteristic curve analysis of all parameters in predicting acute appendicitis

Parameter	Area	Standard error	Asymptomatic 95%CI		p-value	Cutoff value	Sensitivity (%)	Specificity (%)
			Lower bound	Upper bound				
Total count	0.351	0.081	0,191	0.510	0.026^*^	>5300/L	96.5	19
Platelet count	0.448	0.092	0.268	0.628	0.440	>169 × 10^9^/L	96	29
PDW	0.641	0.078	0.488	0.793	0.036^*^	<10.45 fL	73	62
MPV	0.711	0.069	0.576	0.845	0.002^*^	<9.05 fl	57	81
RDW	0.449	0.073	0.306	0.591	0.442	>14.15%	59	48

^*^Significant at 5% level of significance (p<0.05). PDW: platelet distribution width; MPV: mean platelet volume; RDW: red cell distribution width.

## DISCUSSION

Worldwide, acute appendicitis is still a common abdominal emergency. Early and accurate diagnosis of acute appendicitis is required to reduce the morbidity and mortality linked to delayed diagnosis and its complications. Negative appendectomy is also responsible for the loss of important time for medical staff and financial resources, in addition to significant morbidity and death^12^.

The diagnosis of acute appendicitis remains a diagnostic challenge for clinicians despite significant advancements in the diagnostic field and the development of sophisticated examinations. The clinical diagnosis is still debatable because acute appendicitis can occur in a variety of ways, and there are not many trustworthy diagnostic tests available. As was previously said, accessible investigations, such as ultrasonography and computed tomography scans, are costly and time-consuming and require more sophisticated tools and training. Some investigations, however, are neither practical nor readily available^11^.

The diagnosis of acute appendicitis is supported by the use of a variety of clinical grading systems^8^. A clinical scoring system helps improve the first evaluation. The Alvarado scoring system is the most often used of the several scoring systems that are now available. It is supported by historical research, a physical examination, and a few scientific tests. It is a straightforward, cost-effective addition to the clinical diagnosis of acute appendicitis. Although clinical diagnosis is still the mainstay of diagnosing acute appendicitis, their purportedly outstanding results were not always repeatable in everyday situations. Clinical examination precision varied greatly depending on the examiner’s experience, ranging from 71 to 97%. Surgeons have always accepted a 20% probability of unsuccessful appendectomy due to the urgent and life-threatening implications of missing a ruptured appendix^7,16^.

Our study evaluated the diagnostic utility of RDW and platelet indices (i.e., PDW and MPV) in correlation with the clinical diagnosis of acute appendicitis and in predicting perforation. The male-to-female ratio in this study of appendicitis cases was 1.5:1, which indicates a higher male prevalence. This is comparable to the study by Boshnak etal.^4^ (1.1:1) which also found that acute appendicitis is more common in men than women. The age range in this study was 7–72 years. In our study, the ages of patients ranged from 28.04 ± 14.2 years on average. Most of the patients with appendicitis were in their second or third decades of life. This is consistent with Boshnak etal.^4^, Albayrak etal.^2^, and Dinc etal.^9^. No discernible statistical differences between the instances were found.

We observed the cutoff value for the total count of 5300 with a sensitivity of 96.5% and a specificity of 19%. Our study shows statistical significance (p-value=0.026) for leukocyte count, with the mean value of 12211 ± 5611, similar to Kostakis etal.^14^ and Boshnak etal.^4^.

In our study, we found that there was no statistical difference between the acute appendicitis group and the perforated group, which is similar to the results of other studies done by Erdem etal.^10^, Albyarak etal.^2^, and Mehmet etal.^17^. Thus, our analysis found no association of platelet count variations in acute appendicitis. In this study, the mean platelet count in the acute group was 283 ± 70.19, and it was 311 ± 154 with no significance (p=0.44) in the perforated group.

The RDW is an automated measurement of the heterogeneity of red blood cell size and is mostly employed in the differential diagnosis of anemia. It is computed by dividing the mean corpuscular volume by the percentage of the red cell volume’sstandard deviation. A change in RDW level has been observed in a few viral and inflammatory diseases. Inflammatory mediators have been reported to alter the survival of red blood cells in circulation by inhibiting erythrocyte maturation, which causes newer, larger reticulocytes to enter the peripheral circulation and increases RDW. The RDW in our study was found to be 14.71 ± 1.64, which was not significant (i.e., p=0.442 and p>0.05). Similar results were observed in the other studies conducted by Aktimur etal.^1^ and Tanrikulu etal.^21^. However, it was noted that RDW was elevated in the perforated group when compared to the acute group, similar to the study by Boshnak etal.^4^, which shows the high-grade inflammatory pathology of perforated appendicitis, and thus can be used as a predictive indicator.

A decrease in MPV is thought to result from increased sequestration and destruction of activated platelets at the inflammation site, where as an increase in MPV is believed to result from early platelet activation brought on by inflammation and a late rise in the release of young platelets into circulation from the bone marrow^5^. Variations in MPV may be indicative of inflammation, according to numerous studies on non-infectious inflammatory diseases. Studies on acute appendicitis and the function of MPV in its diagnosis have revealed that variations in MPV values, such as higher or lower levels, are acceptable^6,19^. Although, in our study, MPV was found to be statistically significant (i.e., p=0.002 and p<0.05), there was also a decrease in MPV in the perorated group compared to the acute group. This was in concordance with studies conducted by Erdem etal.^10^ and Tanrikulu etal.^21^, thus establishing MPV association in acute appendicitis and predicting perforation. The MPV value evaluated in our study was 9.27 ± 1.06 fl in the acute appendicitis group and 8.47 ± 1.26 fl in the perforated group. We found that MPV is significant (p-value=0.002) in acute appendicitis patients when compared with the perforated group. We obtained a cutoff value of 9.05 fl (p-value=0.002*) for which sensitivity was 57% and specificity was 81%. The results were comparable to those obtained in the study by Aktimur etal.^1^ The quantity of platelets changes during an acute inflammatory process, causing bigger platelets to enter the circulation and, as a result, a spike in subsequent PDW levels. Young platelets enter the peripheral circulation when MPV and PDW are higher than controls. MPV and PDW are both platelet immaturity markers. Our evaluation showed that PDW is statistically significant (i.e., p=0.036 and p<0.05) in the patients of acute appendicitis (11.6 ± 1.91 fl) as compared to the perforated group (10.92 ± 2.47 fl). However, the PDW value was decreased in the perforated group compared to the acute group, which is suggestive of high-grade inflammation. Hence, a decrease in PDW can be used as a prognostic indicator of disease activity in perforated appendicitis. In our study, we obtained a cutoff value of 10.45 fl for PDW, for which sensitivity was 73% and specificity was 62%. This was comparable with the other studies by Dinc etal.^9^ and Boshnak etal.^4^.

## CONCLUSION

Platelet indices and RDW, along with leukocytosis, play an essential role in diagnosing acute and perforated appendicitis and can be used in predicting it preoperatively.

We found that MPV and PDW showed statistical significance in both acute and perforated appendicitis. Also, MPV and PDW were observed to be decreased in perforated appendicitis, which indicates high-grade inflammation, and thus can be used as predictive markers.

RDW showed no significance among both groups. However, perforated appendicitis showed higher RDW than acute appendicitis.

Thus, the value of these can be used as added diagnostic and predictive markers of appendicitis, which are readily available in basic routine CBC tests with no added socio-economic burden to the patients.
